# Distribution and Clinicopathological Features of Mott Cells (Plasma Cells Containing Russell Bodies) in Gastric Cancer: Presence of Mott Cells Is Associated with Favorable Prognosis

**DOI:** 10.3390/jcm13030658

**Published:** 2024-01-23

**Authors:** Go Kobayashi, Takeharu Imai, Kazuhiro Sentani

**Affiliations:** 1 Laboratory of Molecular Pathology, Department of Molecular Biosciences, Radiation Effects Research Foundation, Hiroshima 732-0815, Japan; kobayashi_g@rerf.or.jp; 2 Department of Molecular Pathology, Graduate School of Biomedical and Health Sciences, Hiroshima University, Hiroshima 734-8551, Japan; 3 Department of Surgical Oncology, Graduate School of Medicine Gifu University, Gifu 501-1194, Japan; qqzv23py9@yahoo.co.jp

**Keywords:** gastric cancer, Mott cells, Russell bodies, clinicopathological significance, *Helicobacter pylori*

## Abstract

Gastric cancer (GC) is still one of the leading causes of cancer-related mortality. We previously reported the relationship between histological heterogeneity of tumor cells and molecular features in GC. The tumor microenvironment also has a crucial role in GC progression and therapeutic resistance. In this study, we focused on the tumor microenvironment, especially inflammatory cells in GC. Using GC tissue slides, we investigated the distribution and clinicopathological significance of inflammatory cell counts including eosinophils, neutrophils, lymphocytes, and plasma cells. Additionally, we investigated the relationship between Mott cells (plasma cells containing Russell bodies) and clinicopathological features. In neoplastic gastric mucosa, a high number of plasma cells was associated with low T-grade, early stage, and good prognosis. We then focused on Mott cells and found that their presence in neoplastic gastric mucosa was associated with lower T and N grades, early stage, and Helicobacter pylori infection and was inversely associated with CD44 and EGFR expression. Additionally, the presence of Mott cells was associated with good prognosis in advanced GC and was an independent favorable prognostic predictor. The presence of Mott cells in GC might be one useful prognostic predictor, and Mott cells might have an important role in the carcinogenesis of *H. pylori* infection.

## 1. Introduction

Gastric cancer (GC) is one of the most common human cancers worldwide and still one of the leading causes of cancer-related mortality [1]. The high mortality of GC is mainly due to late diagnosis and poor response to the currently available therapeutic agents. One reason that may explain the poor clinical outcomes of GC is its highly heterogeneous nature [2,3]. Unlike other tumors, GC exhibits diversity in various pathological factors, including histological type, differentiation, stroma, and infiltration patterns. Therefore, an analysis of the heterogeneous nature of GC is necessary.

The development and progression of GC tissue are greatly influenced by the tumor microenvironment [4]. Tumor cells and stromal cells such as inflammatory and immune cells, macrophages, fibroblasts, and endothelial cells are believed to intermingle with each other in a tumor microenvironment [4]. Thus, understanding the different interactions between tumor cells, chronic inflammation, and the immune tumor microenvironment is important to identify new predictive factors for prognosis and therapeutic resistance. In GC, chronic infection by *Helicobacter pylori* (HP) is thought to play a crucial role in the transformation from chronic atrophic gastritis to metaplasia, epithelial dysplasia, and, eventually, adenocarcinoma [5]. In addition, several stromal cells involved in the inflammatory microenvironment of GCs, such as macrophages, lymphocytes, and their products (cytokines), have been reported to promote tumor development at the initiation, progression, and metastasis phases [4,6]. We previously investigated the histological diversity and molecular characteristics of GC and found that GCs with histological diversity on the tumor invasive front have a significantly poorer prognosis and are associated with cancer stem cell-related molecules (CD44, CD133, ALDH1), receptor tyrosine kinase molecules (HER2, EGFR, c-MET), and HP infection [7]. However, these results were focused primarily on tumor cells, and the relationships between tumor microenvironment, clinicopathologic significance, and molecular characteristics have not been examined.

In this study, we first clarified the distribution and clinicopathological significance of inflammatory cell counts including eosinophils, neutrophils, lymphocytes, and plasma cells, which can be detected by hematoxylin and eosin (HE) staining in GC. Additionally, we focused on the accumulation of multiple Russell bodies in “Mott cells”, which are plasma cells in which Russell bodies are occasionally contained. Although there have been some case reports of GC concurrent with Russell bodies in recent years [8,9], the association between clinicopathological significance and the presence of Mott cells in GC continues to remain unclear. Thus, we also investigated the presence and distribution of Mott cells in GC tissue and revealed the relationship between Mott cells and clinicopathologic characteristics for the first time, to our knowledge. We further investigated the association between the presence of Mott cells and survival of patients with GC.

## 2. Materials and Methods

### 2.1. Tissue Samples

All tissue samples were selected from patients diagnosed as having GC who underwent curative surgical resection between 2003 and 2007 at Hiroshima University Hospital or affiliated hospitals (Hiroshima, Japan). Patients with preoperative radiotherapy or chemotherapy and with clinical evidence of distant metastasis were excluded from the study. The distribution and clinicopathological significance of inflammatory cell counts including eosinophils, neutrophils, lymphocytes, and plasma cells were examined using the primary tumor of 229 patients. Survival was analyzed for 103 of the 229 patients because of the availability and reliability of their prognostic information. For further analysis focusing on Mott cells, the number of tumors was increased to a total of 670 primary tumors. Among these 670 patients, survival data were available for 178 patients but not for the other 592 patients. Postoperative follow-up was scheduled every three months after surgery. Serum chemistries, including tumor-specific markers, were performed at every follow-up visit. Chest X-ray or thoraco-abdominal computed tomographic scan was performed every six months. Upper gastrointestinal endoscopy was performed every year. Patients were followed for at least five years until their death or the date of the last documented contact.

### 2.2. Study Design

In this retrospective study, we microscopically counted eosinophils, neutrophils, lymphocytes, plasma cells, and Mott cells in four areas of GC tissue samples, including non-neoplastic gastric mucosa, neoplastic mucosa, the neoplastic invasive front, and the non-neoplastic area around the invasive front on HE-stained slides (Supplementary Figure S1). A range of 3000 μm from the tumor area was used for the evaluation of the non-neoplastic area. First, the number and distribution of eosinophils, neutrophils, lymphocytes, and plasma cells in each of the four areas of GC tissues were investigated. The average number of each type of infiltrating cell per high-power field (HPF) was calculated after counting them in 5 to 10 randomly selected HPFs. Additionally, mean value and standard deviation were calculated in each cell. Second, using the median cut-off values, the count for each cell type was divided into high and low groups, and the relationship between each cell type and clinicopathological significance was evaluated. Third, we microscopically searched the field having the largest number of Mott cells in each of the four areas per low power field (LPF, magnification ×100). We then counted Mott cells per HPF (magnification ×400) in one field of each of the four fields. In addition to the counting of Mott cells, infection by HP was evaluated on the HE-stained slides. One representative tumor block, including the tumor center, invasive front, and tumor-associated non-neoplastic mucosa, was examined from each patient. The count of each cell type was performed independently by 3 investigators (G. K., T. I., and S.K), and when the evaluations differed, a decision was made by consensus while the investigators reviewed the specimen via a multi-head microscope.

Tumor staging was classified according to the latest AJCC/UICC TNM Classification of Malignant Tumors, 8th edition. Because written informed consent was not obtained from the patients, identifying information for all samples was removed before analysis to ensure strict privacy protection. This procedure was in accordance with the Ethical Guidelines for Human Genome/Gene Research enacted by the Japanese Government. This study was approved by the Ethical Committee for Human Genome Research of Hiroshima University, Hiroshima, Japan (No. IRINHI66).

### 2.3. Immunohistochemical Analysis

Immunohistochemical evaluation using representative tumor blocks was performed on the whole sections. For immunostaining of all markers except EGFR, a Dako Envision Kit (Dako Corporation, Carpinteria, CA, USA) was used according to the manufacturer’s recommendations. The antibodies used in the present study and their conditions are shown in Supplementary Table S1. After endogenous peroxidase activity was blocked with 3% H_2_O_2_-methanol for 10 min, sections were incubated with normal goat serum (Dako Corporation) for 20 min to block nonspecific antibody binding sites. The sections were then incubated with the primary antibodies for 1 h at room temperature, followed by incubations with peroxidase-labeled anti-rabbit or mouse IgG for 60 min. For immunostaining of EGFR, a Dako EGFR pharmDx™ assay detection system (Dako Corporation) was used. Staining was completed with a 10 min incubation with the substrate–chromogen solution. The sections were counterstained with 0.1% hematoxylin. Appropriate positive and negative control samples were also stained.

The relationship between Mott cells and various cancer molecules was also analyzed. The cut-off point for antibody reactivity necessary to define a result as positive is shown in Supplementary Table S1. HER2 or c-MET staining was scored as previously reported and was considered to be positive for overexpression if the staining was scored as 2+ or 3+ [10]. MLH1 or MSH2 was considered to be a loss or reduction of nuclear reactivity together with a positive background reaction in non-neoplastic epithelial or stromal cells.

### 2.4. Statistical Analysis

All statistical analyses were performed using SPSS (SPSS Inc., Chicago, IL, USA). Correlations between clinicopathological parameters and the count of eosinophils, neutrophils, lymphocytes, plasma cells, and Mott cells were analyzed using Fisher’s exact test. The Kaplan–Meier method was used to calculate the survival rates of the patients, and differences between groups were examined with a log-rank test. Univariate and multivariate Cox proportional hazards regression analyses were performed to evaluate the associations between clinical covariates and survival. A value of *p* < 0.05 was used to indicate statistical significance.

## 3. Results

### 3.1. Distribution of Eosinophils, Neutrophils, Lymphocytes, and Plasma Cells per Counted Area in Gastric Cancer

**Figure 1:**
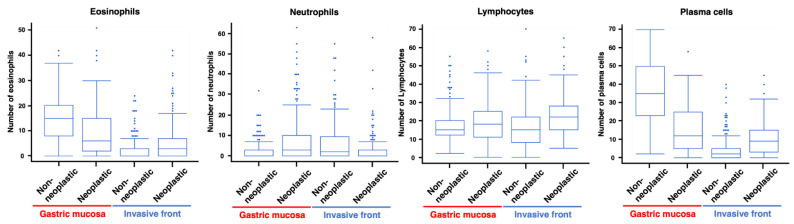
Distribution of each inflammatory cell type per counted area in gastric cancer.

We first counted eosinophils, neutrophils, lymphocytes, and plasma cells in the four areas of the non-neoplastic gastric mucosa, neoplastic mucosa, neoplastic invasive front, and non-neoplastic stroma. The distribution of these cells per HPF in the four areas is summarized in Figure 1. Interestingly, even in the same GC tissue section, the number of inflammatory and immune cells varied depending on the area in which the cells were counted.

**Figure 2:**
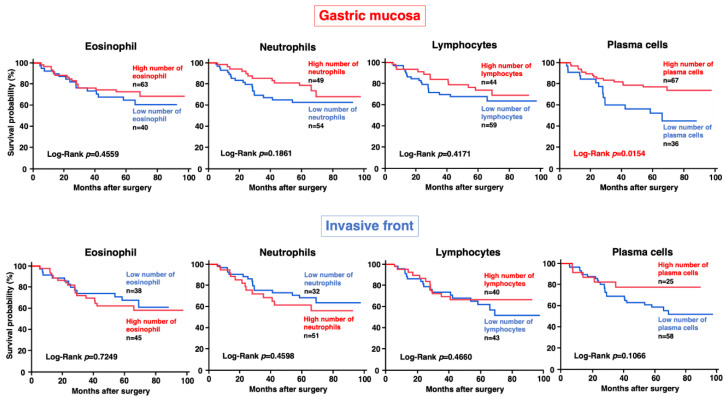
Relationship between counts of each inflammatory cell type in neoplastic gastric mucosa or the invasive front and prognosis.

We then investigated the relationship between inflammatory cell counts in neoplastic areas and clinicopathological features. This revealed that a high number of eosinophils and plasma cells in neoplastic gastric mucosa or the invasive front was associated with low T grade and early stage (Supplementary Tables S2 and S3). We also investigated the association between inflammatory cell counts and prognosis and found that a high number of plasma cells in neoplastic gastric mucosa was associated with a good prognosis (Figure 2).

### 3.2. Distribution of Mott Cells per Counted Area in Gastric Cancer

**Figure 3:**
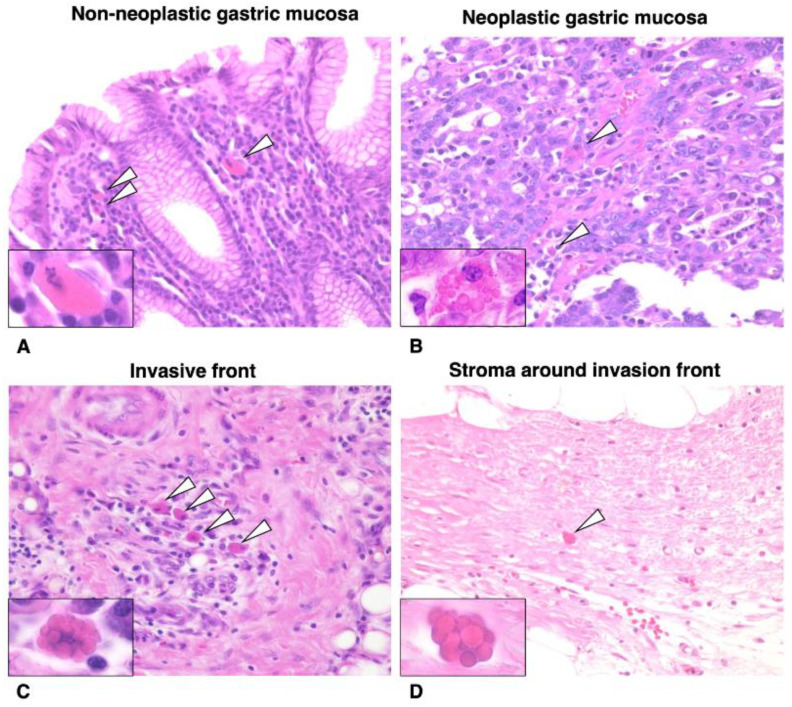
Representative histologic features of Mott cells in (**A**) non-neoplastic gastric mucosa, (**B**) neoplastic gastric mucosa, (**C**) tumor invasive front, and (**D**) stroma around the invasive front.

After revealing that a high number of plasma cells was associated with good prognosis, we realized that in the cases with a high number of plasma cells, these cells occasionally contained Russell bodies. These plasma cells are called Mott cells. Thus, as there was a possibility that Mott cells might be one important factor for predicting GC prognosis, we focused on the presence of Mott cells in GC tissues. The representative histologic features of Mott cells in the four areas are shown in Figure 3A–D. Additionally, the distribution of Mott cells per HPF in GC are summarized in Table 1. The presence of Mott cells was found in all four areas as follows: non-neoplastic gastric mucosa (203/550 cases, 37%), neoplastic mucosa (153/670 cases, 23%), neoplastic area of the invasive front (18/518 cases, 3.5%), and non-neoplastic stroma around the invasive front (2/517 cases, 0.4%). In all four areas, we more frequently found only one Mott cell (low group) compared with finding two or more Mott cells (high group) (Table 1).

### 3.3. Relationship between Presence of Mott Cells in Neoplastic Gastric Mucosa and Clinicopathological Characteristics

As Mott cells were occasionally observed in gastric mucosa but were rarely seen in the invasive front, we therefore examined the relationship between the presence of Mott cells in neoplastic gastric mucosa and clinicopathological characteristics. The presence of Mott cells was significantly associated with T classification (*p* = 0.0014), N classification (*p* = 0.0378), stage (*p* = 0.0023), and HP infection (*p* < 0.0001) (Table 2) and was frequently observed in T1 cases, N0 cases, stage I cases, and cases positive for HP infection. Moreover, the presence of Mott cells in non-neoplastic gastric mucosa was also significantly associated with T classification (*p* = 0.0005), stage (*p* = 0.0435), and HP infection (*p* = 0.0002) (Supplementary Table S4). We also used a previous cohort of 116 GC cases to investigate the relationship between Mott cells and various cancer-related molecules in the neoplastic gastric mucosa. The presence of Mott cells was significantly inversely associated with the expression of EGFR (*p* = 0.0352) and CD44 (*p* = 0.0429) (Table 3).

### 3.4. Association between Presence of Mott Cells in Neoplastic Gastric Mucosa and Survival of 178 Patients with GC

**Figure 4:**
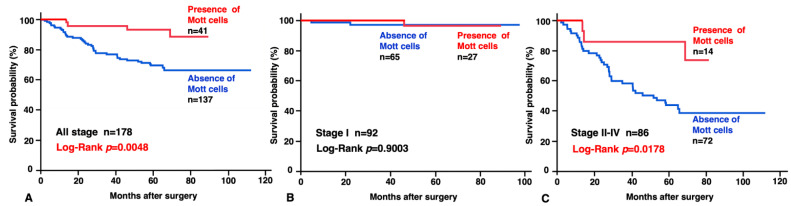
Relationship between the presence of Mott cells in neoplastic gastric mucosa. Overall survival of the patients with (**A**) all stages (n = 178), (**B**) stage I, and (**C**) stages II/III/IV, respectively.

Finally, we investigated the relationship between the presence of Mott cells in neoplastic gastric mucosa and survival of patients with GC. Kaplan–Meier analysis showed the presence of Mott cells to be significantly associated with a favorable prognosis (*p* = 0.0048; Figure 4A). In addition, we analyzed the prognostic effect of Mott cells in the stage I and the stage II/III/IV tumors. Although the presence of Mott cells was not associated with survival in patients with GC stage I (Figure 4B), in the patients with GC stages II/III/IV, Kaplan–Meier analysis showed better survival in the patients with than without Mott cells (*p* = 0.0178; Figure 4C). Moreover, uni- and multivariate Cox proportional hazards analyses showed that the presence of Mott cells was an independent favorable prognostic predictor for the survival of patients with GC (Table 4).

## 4. Discussion

Previously, we showed that histological diversity has an important role in the progression of GC and its molecular characteristics [7]. In the present study, we first focused on the tumor microenvironment and investigated the relationship between inflammatory cell counts and clinicopathological characteristics. As a result, a high number of eosinophils in the neoplastic region was associated with a low T grade and early stage. Indeed, high eosinophil counts are reported to be associated with a significantly favorable prognosis in several cancers including colon, breast, and GC [11,12,13,14]. Previous studies have also shown that intense lymphocytic infiltration in GC patients was associated with a favorable prognosis [15,16] and, especially, that high levels of B-cell infiltration were associated with low lymph node involvement, low TNM stage, and a favorable prognosis [17]. In the present study, patients with plasma cell infiltrations in the tumor region had a better prognosis. Indeed, previous studies have shown that tumor-infiltrating plasma cells were associated with an improved prognosis in several cancers including GC [18,19,20]. Interestingly, plasma cells containing Russell bodies, i.e., Mott cells, were occasionally observed in the gastric mucosa in patients with a high number of plasma cells. Because the clinicopathological significance and molecular characteristics of Mott cells in GC remain to be elucidated, and we thus conducted a detailed analysis of these cells.

Russell bodies are eosinophilic globules and structures including spherical immunoglobulin derived from plasma cells. These were first described by Russell in 1890 [21]. Russell bodies represent a general cellular response to the accumulation of abundant, non-degradable immunoglobulin [22]. It has been speculated that chemokine production associated with chronic inflammation leads to overstimulation of plasma cells and the resulting formation of Russell bodies [8,23,24]. Thus, Russell bodies can appear in various organs and different diseases, either benign or malignant [22]. In 1998, Tazawa and Tsutsumi reported a case of plasma cells containing Russell bodies in the gastric mucosa of a patient with HP infection [25]. They named the pathological condition “Russell body gastritis (RBG)”, which is now considered to be a subtype of chronic gastritis [26]. In recent years, Altindag et al. summarized 12 case reports of RBG and revealed that HP was detected in half of the cases, with RBG being concurrent with GC in one case [27]. Although the percentage of RBG appearing with GC is not well known, among the present 670 cases, we suspected that only one case, which had 11 Mott cells in the gastric mucosa on HPF counting, was RBG concurrent with GC. Therefore, we consider this case of RBG concurrent with GC to be rare.

With regard to the relationship between Russell bodies and GC, Johansen and Sikjär reported on the diagnostic significance of Russell bodies in endoscopic gastric biopsies in 1977 [28]. According to these authors, Russell bodies were found in 38% of non-neoplastic gastric mucosa and were associated with early GC [28]. Similar to their results, we found Mott cells in 37% of the non-neoplastic gastric mucosa in the present study. Moreover, we found Mott cells in 23% of the neoplastic gastric mucosa, and their presence was associated with T1, N0, and stage I cancer. The presence of Mott cells in neoplastic gastric mucosa was also associated with a good prognosis in stage II to IV GC cases and was an independent predictor of better prognosis. Although Mott cells are commonly observed in hematopoietic malignancies such as plasma cell myeloma, MALT lymphoma, plasmacytoma, or lymphoplasmacytic lymphoma [24,29,30], their association with epithelial tumors, including GC, remains unclear. The exact pathogenic mechanism leading to the development of Mott cells is not fully understood; however, they are often formed in response to immune or inflammatory reactions [23,24,31,32]. Given the well-established association between an increased presence of immune cells and a favorable prognosis in GC [15,16,17,20,33,34], Mott cells might be induced by the immune or inflammatory response in tumor microenvironments of GC, resulting in the correlation between the presence of Mott cells and a favorable prognosis. Previously, we demonstrated that histological diversity of tumor cells at the tumor invasive front shows a significantly poor prognosis. Therefore, in addition to the morphological diversity of tumor cells, the evaluation of the tumor stroma, especially for Mott cells, may help in assessing the prognosis of advanced GC. Moreover, Takahashi et al. suggested an association between the production of chemokines in tumor cells and the development of Russell bodies [29]. These findings indicate that the interaction between Mott cells and tumor cells might have an important role in tumor progression in GC and that evaluating the presence or absence of Mott cells might be useful for predicting the progression of GC.

Although previous studies showed cases of RBG to be associated with infection by HP [25,27], the relationship between Russell bodies and HP in GC remains unclear. In the present study, the presence of Mott cells was significantly associated with infection by HP in both neoplastic and non-neoplastic areas of the gastric mucosa. HP is well known to be associated with the development of GC. Two major factors of HP virulence are cytotoxin-associated gene A (CagA) and vacuolating cytotoxin A (VacA) [35]. CagA has been shown to be closely associated with higher grades of gastric mucosal inflammation as well as severe atrophic gastritis and has an important role in the development of GC [36]. VacA also induces gastric inflammation and contributes to GC carcinogenesis [37]. Therefore, there might be an interaction between these virulence factors, the production of chemokines, and the formation of Mott cells in HP infection, and they might support the processes of GC carcinogenesis. In addition, we revealed that the presence of Mott cells was inversely associated with cancer stem cell markers such as CD44 or tyrosine kinase molecules such as EGFR. Although further study is needed, the presence or absence of Mott cells might be one of the important factors of cancer cell biology in GC.

This study was associated with some limitations, including its retrospective nature. Therefore, a prospective series is necessary. In addition, since the number of stage II-IV patients was smaller than in stage I patients, a further extensive study will be required. Second, although we revealed the association between the presence of Mott cells and HP infection, the detailed molecular mechanisms remain unclear. Further studies are necessary to elucidate the molecular activity of Mott cells in tumor cell biology. Third, because we diagnosed HP infection from HE staining, it is possible that HP infection and the presence of Mott cells could be found more frequently using other methods, including immunohistochemistry, periodic acid-Schiff staining, urea breath tests, antibody tests, and rapid urease tests.

## 5. Conclusions

In summary, we investigated the distribution and clinicopathological significance of inflammatory cell counts and revealed that the cases with a high number of plasma cells had a favorable prognosis. Among the cases of a high number of plasma cells, we focused on the presence or absence of Mott cells. The relationship between clinicopathological significance and the presence of Mott cells revealed that Mott cells were significantly associated with less pathologically aggressive features and HP infection, suggesting that interactions between Mott cells and tumor cells might be important factors in carcinogenesis of GC. Additionally, the presence of Mott cells was associated with a favorable prognosis in advanced GC cases, suggesting that evaluating the presence or absence of Mott cells might be one of the useful factors for appropriate patient management.

## Figures and Tables

**Table 1:** Distribution of Mott cells per counted area in gastric cancer.

		Number of Mott Cells/HPF				
		Non-Presence	Presence			
			Low	High		
		0	1	2–5	6–10	11–
Gastric mucosa						
	Non-neoplastic (n = 554)	351 (63)	203 (37)			
			130 (23.6)	66 (12)	5 (1)	2 (0.4)
	Neoplastic (n = 670)	517 (77)	153 (23)			
			122 (18)	30(4.8)	0 (0)	1 (0.2)
Invasive front						
	Neoplastic area (n = 518)	500 (96.5)	18 (3.5)			
			14 (2.7)	4 (0.8)	0 (0)	0 (0)
	Non-neoplastic stroma (n = 517)	515 (99.6)	2 (0.4)			
			1 (0.2)	1 (0.2)	0 (0)	0 (0)

**Table 2:** Relationship between presence of Mott cells in neoplastic gastric mucosa and clinicopathological characteristics.

		Mott Cell		*p* Value
		Presence n = 153	Non-Presence n = 517	
Age				
	<65 (n = 326) ≥65 (n = 344)	75 (23%) 78 (22%)	251 266	0.9186
Sex				
	Male (n = 420) Female (n = 250)	88 (20%) 65 (26%)	332 185	0.1345
Histologic classification				
	Differentiated (n = 361) Un-differentiated (n = 309)	75 (21%) 78 (25%)	286 231	0.1702
T classification				
	T1 (n = 314) T2/T3/T4 (n = 356)	89 (28%) 64 (18%)	225 292	0.0014
N classification				
	N0 (n = 385) N1/N2/N3 (n = 285)	99 (26%) 54 (19%)	286 231	0.0378
Stage				
	I (n = 380) II/III/IV (n = 290)	103 (27%) 50 (17%)	277 240	0.0023
*H. pylori*				
	Negative (n = 566) Positive (n = 104; 15%)	108 (19%) 45 (43%)	458 59	<0.0001

*p* values were calculated using Fisher’s exact test. Bold values indicate statistical significance (*p* < 0.05).

**Table 3:** Relationship between presence of Mott cells in neoplastic gastric mucosa and representative molecular markers.

		Mott Cell		*p* Value
		Presence n = 25	Non-Presence n = 91	
PD-L1	Negative (n = 98) Positive (n = 18)	21 (79%) 4 (22%)	77 14	1.0000
MLH1	Negative (n = 26) Positive (n = 90)	4 (15%) 21 (23%)	22 69	0.5884
MSH2	Negative (n = 5) Positive (n = 111)	2 (40%) 23 (21%)	3 88	0.2935
HER2	Negative (n = 88) Positive (n = 28)	18 (21%) 7 (25%)	70 21	0.6058
**EGFR**	**Negative** (n = 87) Positive (n = 29)	23 (26%) 2 (22%)	64 27	**0.0352**
c-MET	Negative (n = 96) Positive (n = 20)	23 (24%) 2 (10%)	73 18	0.2361
**CD44**	**Negative** (n = 62) Positive (n = 54)	18 (29%) 7 (13%)	44 47	**0.0429**
ALDH1	Negative (n = 55) Positive (n = 71)	12 (27%) 13 (18%)	33 58	0.3553
CD133	Negative (n = 96) Positive (n = 20)	24 (25%) 1 (5%)	72 19	0.0698
p53	Negative (n = 71) Positive (n = 45)	17 (24%) 8 (18%)	54 37	0.4313

*p* values were calculated using Fisher’s exact test. Bold values indicate statistical significance (*p* < 0.05).

**Table 4:** Univariate and multivariate Cox regression analysis of existence of Mott cells and survival.

Characteristic	Univariate Analysis		Multivariate Analysis	
	HR (95%CI)	*p* Value	HR (95%CI)	*p* Value
Age				
<65	1 (Ref.)			
≧65	0.94 (0.51–1.73)	0.9446		
Sex				
Male	1 (Ref.)			
Female	1.01 (0.51–1.91)	0.9632		
Histological classification				
Differentiated	1 (Ref.)			
Un-differentiated	1.34 (0.73–2.63)	0.3275		
Stage				
I	1 (Ref.)		1 (Ref.)	**<0.0001**
II/III/IV	1.06 (0.57–2.08)	0.8400	20.78 (6.42–67.24)	
Mott cell				
Non-presence	1 (Ref.)		1 (Ref.)	
Presence	0.26 (0.06–0.74)	**0.0082**	0.34 (0.12–0.96)	**0.0423**

Bold values indicate statistical significance (*p* < 0.05).

## Data Availability

All data generated or analyzed during this study are included in this published article.

## Supplementary Materials

The following supporting information can be downloaded at: https://www.mdpi.com/article/10.3390/jcm13030658/s1, Figure S1: Four areas (non-neoplastic gastric mucosa, neoplastic mucosa, neoplastic invasive front, and non-neoplastic stroma) for evaluating inflammatory cells on HE slides of GC tissues; Table S1: Information on antibodies used in this study and their rates of positivity; Table S2: Relationship between high or low presence of blood cells and clinicopathological parameters in the neoplastic area of gastric mucosa; Table S3: Relationship between high or low presence of blood cells and clinicopathological parameters in the neoplastic area of the invasive front; and Table S4: Relationship between presence of Mott cells in non-neoplastic gastric mucosa and clinicopathological characteristics.
