# Supplementary material for: Distribution and Clinicopathological Features of Mott Cells (Plasma Cells Containing Russell Bodies) in Gastric Cancer: Presence of Mott Cells Is Associated with Favorable Prognosis
Source: J Clin Med. 2024 Jan 23;13(3):658. doi: 10.3390/jcm13030658 (PMC10856670; doi:10.3390/jcm13030658)
Supplement: Supplementary file 1 [file jcm-13-00658-s001.zip › jcm-2749382-supplementary.pdf]

Supplementary Materials

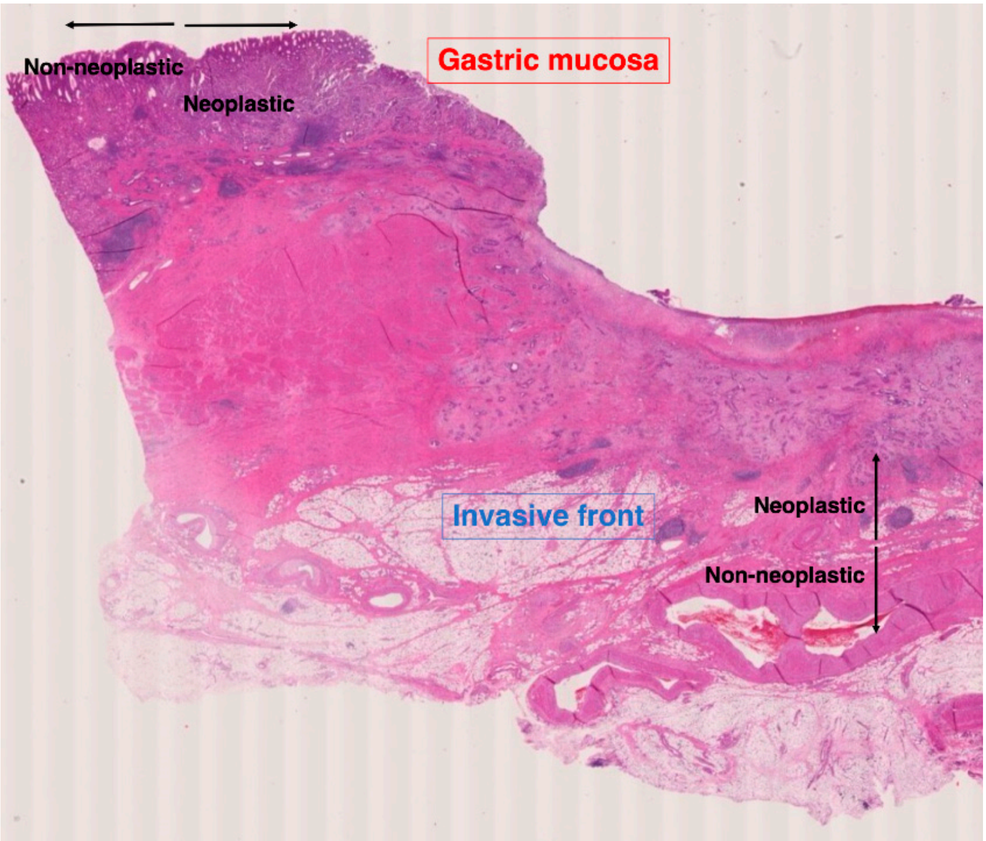

Figure S1. Four areas (non-neoplastic gastric mucosa, neoplastic mucosa, neoplastic invasive front, and non-neoplastic stroma) for evaluating inflammatory cells on HE slides of GC tissues.

Table S1. Information on antibodies used in this study and their rates of positivity.

Antibody	Clone	Dilution	Source	Cutoff-point	Positivity (%)
PD-L1	E1L3N	1: 200	Cell Signaling Technology	5%≤	18/116 (16%)
MLH1	ES05	1: 50	DAKO	Loss or reduction	26/116 (10%)
MSH2	FE11	1: 50	DAKO	Loss or reduction	5/116 (4%)
HER2	SV2-61γ	Diluted	Nichirei	2+, 3+	25/116 (23%)
EGFR	2-18C9	Diluted	DAKO	10%≤	29/116 (19%)
c-MET	SP44	Diluted	Ventana Medical Systems	2+, 3+	20/116 (19%)
CD44	DF1485	1: 50	Novocastra	10%≤	54/116 (45%)
ALDH1	44/ALDH	1: 200	BD Biosciences	10%≤	71/116 (63%)
CD133	AC133	1: 100	Miltenyi Biotec	10%≤	20/116 (17%)
p53	DO-7	1: 50	Novocastra	50%≤	45/116 (21%)

Table S2. Relationship between high or low presence of blood cells and clinicopathological parameters in the neoplastic area of gastric mucosa.

	Eosinophils			Neutrophils			Lymphocytes			Plasma cells		
	High	Low	*P*	High	low	*P*	High	low	*P*	High	low	*P*
Age												
<65	69 (61%)	44	**0.0081**	56 (50%)	57	1.0000	54 (48%)	59	02889	52 (46%)	61	1.0000
>65	50 (42%)	66		57 (49%)	59		47 (41%)	69		53 (46%)	63	
Sex												
Female	57 (55%)	47	0.5067	57 (55%)	47	0.1455	53 (51%)	51	0.0623	48 (46%)	56	1.0000
Man	62 (50%)	63		56 (45%)	69		48 (38%)	77		57 (46%)	68	

Histology												
tub/pap	62 (53%)	55	0.8947	63 (54%)	54	0.1848	44 (38%)	73	**0.0335**	57 (49%)	60	0.4242
por/sig	57 (52%)	53		49 (45%)	61		57 (52%)	53		47 (43%)	63	
T grade												
Tis/T1	64 (67%)	32	**0.0002**	51 (53%)	45	0.3309	37 (39%)	59	0.1777	53 (55%)	43	**0.0221**
T2/3/4	55 (41%)	78		62 (47%)	71		64 (48%)	69		52 (39%)	81	
N grade												
N0	69 (56%)	55	0.2854	67 (54%)	57	0.1105	53 (43%)	71	0.5910	58 (47%)	66	0.8934
N1/2/3	49 (48%)	53		44 (43%)	58		48 (47%)	54		46 (45%)	56	
Stage												
Stage 0/1	70 (60%)	47	**0.0174**	66 (56%)	51	**0.00345**	46 (39%)	71	0.1452	51 (52%)	56	0.0633
Stage 2/3/4	49 (44%)	63		47 (42%)	65		55 (49%)	57		44 (39%)	68	

p values were calculated using Fisher's exact test.
Bold values indicate statistical significance (p<0.05)

Table S3. Relationship between high or low presence of blood cells and clinicopathological parameters in the neoplastic area of the invasive front.

	**Eosinophils**			**Neutrophils**			**Lymphocytes**			Plasma cells		
	**High (%)**	**Low**	***P***	**High**	**low**	***P***	**High**	low	*P*	High	Low	*P*
Age												
<65	34 (45%)	42	1.0000	32 (42%)	44	0.2745	36 (47%)	40	04333	29 (38%)	47	0.3424
>65	39 (44%)	49		45 (51%)	43		36 (41%)	52		41 (47%)	47	
Sex												
Female	36 (47%)	40	0.5309	36 (47%)	40	1.0000	37 (49%)	39	0.2725	29 (38%)	47	0.3424
Man	37 (42%)	51		41 (47%)	47		35 (40%)	53		41 (47%)	47	
Histology												
tub/pap	38 (38%)	41	0.4341	47 (59%)	32	**0.0029**	28 (35%)	51	0.0576	39 (49%)	40	0.0837
por/sig	35 (42%)	49		30 (36%)	54		43 (52%)	41		30 (36%)	54	
T grade												
Tis/T1	24 (63%)	14	**0.0096**	51 (53%)	45	0.3309	18 (43%)	20	0.7100	22 (58%)	16	**0.0395**
T2/3/4	49 (39%)	77		62 (47%)	71		54 (43%)	72		48 (38%)	78	
N grade												
N0	31 (48%)	34	0.6324	33 (51%)	32	0.5207	33 (51%)	32	0.1961	31 (48%)	34	0.4196
N1/2/3	42 (44%)	54		43 (45%)	53		38 (40%)	58		39 (41%)	57	
Stage												
Stage 0/1	33 (58%)	24	**0.0137**	28 (49%)	29	0.7435	28 (49%)	29	0.4089	31 (54%)	26	**0.0318**
Stage 2/3/4	40 (37%)	67		49 (46%)	58		44 (41%)	63		39 (36%)	68	

p values were calculated using Fisher's exact test.
Bold values indicate statistical significance (p<0.05)

Table S4. Relationship between presence of Mott cells in non-neoplastic gastric mucosa and clinicopathological characteristics.

		Mott cell		
		Existence n=203	Non-existence n=351	*p* value
**Age**				
	< 65 (n=274)	106 (39%)	168	0.3234
	≥ 65 (n=280)	97 (35%)	183	
Sex				
	Male (n=352)	120 (34%)	232	0.0999

	Female (n=202)	83 (41%)	119	
Histologic classification				
	Differentiated (n=307)	108 (35%)	199	0.4254
	Un-differentiated (n=247)	95 (38%)	95	
**T classification**				
	**T1** (n=293)	127 (43%)	166	**0.0005**
	T2/T3/T4 (n=261)	76 (29%)	185	
N classification				
	N0 (n=357)	139 (38%)	218	0.1316
	N1/N2/N3 (n=197)	64 (32%)	133	
**Stage**				
	**I** (n=352)	140 (40%)	212	**0.0435**
	II/III/IV (n=202)	63 (31%)	139	
**H. pylori**				
	Negative (n=454)	150 (33%)	304	**0.0002**
	**Positive** (n=100)	53 (53%)	47	

p values were calculated using Fisher's exact test.
Bold values indicate statistical significance ($p<0.05$)
